# p.Phe508del-CFTR Trafficking: A Protein Quality Control Perspective Through UPR, UPS, and Autophagy

**DOI:** 10.3390/ijms26083623

**Published:** 2025-04-11

**Authors:** Pascal Trouvé, Claude Férec

**Affiliations:** Univ Brest, Inserm, EFS, UMR 1078, 22 Avenue Camille Desmoulins, F-29200 Brest, France; claude.ferec@univ-brest.fr

**Keywords:** conventional protein secretion (CPS), unconventional protein secretion (UPS), unfolded protein response (UPR), autophagy, p.Phe508del-CFTR, cystic fibrosis (CF)

## Abstract

Cystic fibrosis (CF) is a genetic disease due to mutations in the cystic fibrosis transmembrane conductance regulator (*CFTR*) gene. The most frequent mutation (p.Phe508del) results in a misfolded protein (p.Phe508del-CFTR) with an altered transport to the membrane of the cells via the conventional protein secretion (CPS) pathway. Nevertheless, it can use unconventional protein secretion (UPS). Indeed, p.Phe508del-CFTR forms a complex with GRASP55 to assist its direct trafficking from the endoplasmic reticulum to the plasma membrane. While GRASP55 is a key player of UPS, it is also a key player of stress-induced autophagy. In parallel, the unfolded protein response (UPR), which is activated in the presence of misfolded proteins, is tightly linked to UPS and autophagy through the key effectors IRE1, PERK, and ATF6. A better understanding of how UPS, UPR, and stress-induced autophagy interact to manage protein trafficking in CF and other conditions could lead to novel therapeutic strategies. By enhancing or modulating these pathways, it may be possible to increase p.Phe508del-CFTR surface expression. In summary, this review highlights the critical roles of UPS- and UPR-induced autophagy in managing protein transport, offering new perspectives for therapeutic approaches.

## 1. Introduction

The trafficking of membrane protein is a complex process by which proteins are functional at the right time and place. Disruptions in this intricate system can lead to various diseases or exacerbate their pathogenicity. Among them is cystic fibrosis (CF). It is a lethal genetic disease primarily affecting the Caucasian population, caused by mutations in the *CFTR* (cystic fibrosis transmembrane conductance regulator) gene [1,2]. The most frequent variant of *CFTR* is Phe508del (c.1521_1523delCTT), which encodes the p.Phe508del-CFTR protein, known to exhibit trafficking defects. The present review aims to explain how it reaches the plasma membrane, focusing on unconventional protein export mechanisms and their link to autophagy and cellular stress. We propose that this underexplored mechanism may provide new therapeutic perspectives for managing CF and other diseases involving protein-folding defects.

CFTR (ABCC7) is a transmembrane protein expressed in the apical membrane of epithelial cells, where it facilitates the transport of chloride (Cl^−^) and bicarbonate (HCO_3_^−^) ions [3,4,5]. Its structure comprises two transmembrane domains (TMDs) and two cytosolic ATP-binding domains (NBDs), consisting of two homologous halves, with TMD1 linked to NBD1 and TMD2 linked to NBD2. The CFTR protein further possesses a unique regulatory domain (R) linking TMD1/NBD1 to TMD2/NBD2 [6]. Wild-type CFTR exists in three electrophoretically distinguishable forms: band A (non-glycosylated, 127 KDa), band B (core-glycosylated, 130 KDa), and band C (complex-glycosylated, mature CFTR, 170 KDa) [7]. These glycosylation states correspond to CFTR’s journey through the secretory pathway.

The p.Phe508del mutations, a deletion of phenylalanine at position 508, results in a misfolded protein retained within the endoplasmic reticulum (ER) and rapid degradation by ER-associated degradation (ERAD) [8,9]. Chaperone machinery that assists CFTR folding includes cytosolic heat shock proteins, such as Hsp70, Hsp90, and Hsp40, and the ER resident calnexin [10,11,12,13]. Electrophoretic analysis reveals that band C, representing the mature form of CFTR, is absent in p.Phe508del-CFTR, which remains in its core-glycosylated form. Only a negligible amount of this mutant protein is expressed at the plasma membrane.

Interestingly, some evidence suggests that p.Phe508del-CFTR can bypass the conventional trafficking pathway and reach the plasma membrane via unconventional protein secretion (UPS), which is a stress-induced pathway [14,15]. This mechanism involves the Golgi reassembly stacking protein (GRASP55) [15,16] and likely involves vesicular components associated with secretory autophagy, a non-degradative autophagic process facilitating cargo delivery to the plasma membrane. The secretory autophagic process assembles core autophagic machinery proteins to trap the N-amino terminal leader sequence of proteins by coordinating Golgi re-assembly and stacking proteins (GRASPs), which are biomarkers of secretory autophagy [17,18]. Unlike canonical autophagy, which primarily degrades intracellular components, secretory autophagy employs core autophagic machinery that can transport p.Phe508del-CFTR. The process is triggered by cellular stressors, including inflammation and ER stress, which activate the unfolded protein response (UPR) to alleviate the protein-folding burden in the ER [19].

Although still debated because the mutated CFTR is rapidly degraded, the retention and/or accumulation of the p.Phe508del-CFTR protein within the ER likely induces an ER stress that, in combination with other factors such as inflammation [19], triggers the unfolded protein response (UPR) [20,21]. The UPR is a normal physiological recovery process aimed at regulating the protein load in the ER and alleviating the cellular stress. GRP78/BiP triggers the UPR when it binds to the unfolded protein [22,23]. This binding leads to its dissociation from the three main effectors of UPR, which are inositol-requiring enzyme 1α (IRE1α) [24,25], protein kinase R (PKR)-like endoplasmic reticulum kinase (PERK) [26], and activating transcription factor 6 (ATF6) [27,28,29]. These effectors then activate the transcription of genes encoding molecular chaperones, folding catalysts and proteins involved in the ERAD. They also decrease the global synthesis of proteins to avoid an overload of the ER [30]. Interestingly, ATF6 is known to decrease *CFTR* expression [31,32]. It also triggers the degradative autophagy.

We propose here that the binding of p.Phe508del-CFTR to GRASP55 may facilitate its escape from ER quality control, enabling its transport to the plasma membrane via secretory autophagy when the UPR is triggered.

Protein quality control is an essential process involving sophisticated mechanisms such as the ER stress response (UPR), the ubiquitin–proteasome system, and autophagy. These signaling pathways, although distinct, are closely interconnected and play a crucial role in managing misfolded or damaged proteins. In this context, p.Phe508del-CFTR constitutes a paradigmatic model for studying the impact of quality control defects on disease pathogenesis. This review aims to explore in depth the mechanisms of UPR, UPS, and autophagy, highlighting their role in the management of p.Phe508del-CFTR and potential therapeutic implications. Therefore, this review presents the current understanding of the synthesis, degradation, and membrane export of p.Phe508del-CFTR, emphasizing the interplay between GRASP55, secretory autophagy, and cellular stress responses. By elucidating these interconnected mechanisms, we aim to provide insights into how unconventional secretion pathways can be leveraged therapeutically. A comprehensive diagram summarizing these processes is proposed, linking cellular stress to protein trafficking and suggesting novel approaches for addressing CF and other protein-folding disorders.

## 2. Biogenesis of CFTR and Differences with the Biogenesis of p.Phe508del-CFTR

CFTR follows the conventional endoplasmic reticulum (ER)–Golgi-mediated pathway [33]. In the conventional pathway, the precursor protein can be translocated during or after its synthesis at the ribosome, and the co-translational [34] and posttranslational [35] translocation are distinguished. The folding of CFTR occurs via co- and post-translational mechanisms [36,37]. CFTR biogenesis initiates in cytosolic ribosomes, which are directed to the ER membrane translocon (Sec61 complex) by the signal recognition particle (SRP) via its receptor [36,38,39].

CFTR employs two redundant pathways at the ER membrane to ensure correct N-terminal topology. In one, TMD1 initiates translocation and spans the membrane, with transmembrane TMD2 later terminating translocation co-translationally [36]. If TDM1 fails, TDM2 serves as a secondary signal anchor to correctly orient the protein. Co-translational translocation moves the chain from the N to the C terminus in the ER lumen, while posttranslational translocation moves it from the C to the N terminus. Co-translational folding produces cytosolic NBD1 and R domain before membrane-spanning domain MSD2 integrates into the ER membrane [40].

**Figure 1:**
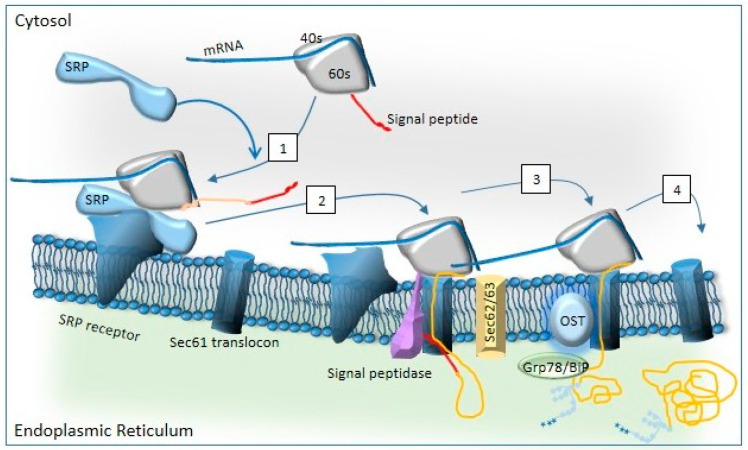
Co-translational translocation via the Sec61 translocon. Proteins are targeted to the ER membrane by the binding of a signal recognition particle (SRP) to the signal sequence (step 1). SRP binding pauses the protein synthesis to keep the polypeptide chain in a conformation suitable for transport. At the ER membrane, SRP binds to its receptor. The complex is then transferred to the Sec61 translocon, and the ribosome interaction with the translocon reinitiates translation and induces conformational changes within Sec61α (step 2). In the case of a weak hydrophobic SP, the protein needs the help of accessory proteins such as Sec62/Sec63 for protein translocation. The signal peptide (SP) is then cleaved by the signal peptidase complex and the protein is glycosylated by the oligosaccharyl transferase complex (OST). BiP guarantees the effective translocation of proteins via the Sec61 translocon and also facilitates their proper folding (step 3). The new protein is then released inside the ER (step 4).

Each CFTR domain folds independently, achieving native conformation through co- and post-translational processes. Interdomain interactions are critical for the final structure, as mutations in transmembrane domains can disrupt the folding of NBDs and vice versa. The Sec61 translocon is a multi-protein channel embedded in the ER membrane and facilitates CFTR’s entry into the ER (Figure 1) [41].

This complex includes Sec61α, Sec61β, and Sec61γ subunits, along with accessory proteins like Sec62, Sec63, and the TRAP (translocon-associated protein) complex, which aid in ribosome recruitment and protein translocation [42]. Hsp70 and its ER-resident counterpart BiP (Grp78) prevent substrate misfolding and assist in translocation [43]. Whereas Hsp70 prevents the misfolding of translocated substrates in the ER lumen, BiP directly acts in the translocation process, as described below [41,43,44,45].

**Figure 2:**
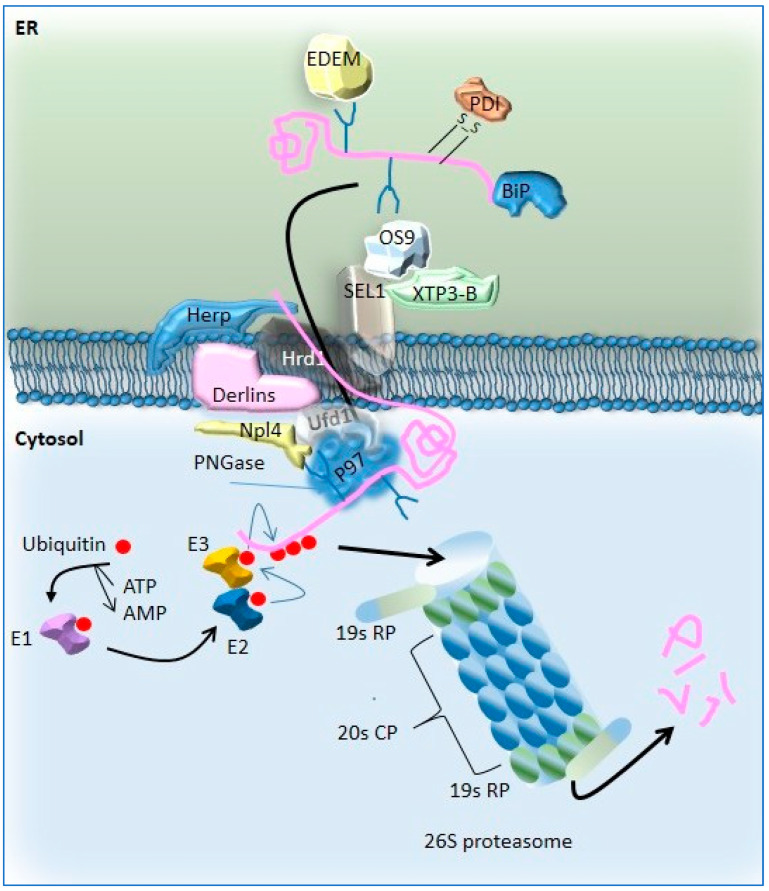
Overview of the ERAD process. Unfolded and misfolded proteins are recognized by chaperones and chaperone-like lectins in the lumen of the ER. Sugar chains are recognized, and unfolded and misfolded proteins are localized in the ERAD complex and are retrotranslocated to the cytoplasm using the energy from the p97 complex. In the cytoplasm, the substrate is ubiquitinated and further degraded by the 26S proteasome. Ubiquitination process: E1 enzymes activate ubiquitin molecules in an ATP-dependent manner and transfer ubiquitin to the E2 ubiquitin-conjugated enzyme. E2 and its linked ubiquitin binds to E3, which binds to the substrate and transfers ubiquitin. Proteasome structure: The 26S proteasome is a protease enzyme complex in which protein hydrolysis occurs. 26S proteasome is composed of 20S proteasome (core protein, CP) and two 19S regulatory proteins (RPs). (Herp: homocysteine-induced endoplasmic reticulum protein; SEL1: suppressor/enhancer of lin-12-like; XTP3-B: XTP3-transactivated gene B; OS-9: osteosarcoma-9; ATP: adenosine triphosphate; Derlins: Der-like domain-containing family proteins; Ufd1: ubiquitin-fusion degradation protein 1; Npl4: nucleoprotein locates protein 4; E3: E3 ubiquitin ligase; ATP: adenosine triphosphate; AMP: adenosine monophosphate).

Oligosaccharyltransferase (OST) initiates N-linked glycosylation of specific asparagine residues, then binds to the ribosome–Sec61–TRAP complex [46]. Interestingly, the ER translocon is involved in the UPR [47]. Glycosylation intermediates (Glc3Man9GlcNAc2, Glc2Man9GlcNAc2, Glc1Man9GlcNAc2) are trimmed by glucosidases I and II, enabling lectin-like proteins to direct CFTR into the calnexin/calreticulin (CNX/CRT) cycle, where it undergoes further folding and quality control [48,49,50]. Protein disulfide isomerase (PDI) catalyzes disulfide bond formation to stabilize CFTR [50,51]. After exiting the CNX/CRT cycle, CFTR can undergo one of three fates [52]: correctly folded CFTR progresses to the Golgi for further glycosylation, incompletely folded CFTR re-enters the CNX/CRT cycle via UDP–glycoprotein glycosyltransferase (UGT) [53,54,55], or misfolded CFTR is degraded via ER-associated degradation (ERAD) [56,57,58,59,60]. ERAD [56,57] is a pre-Golgi proteolysis pathway targeting misfolded proteins [58,59,60]. It relies on the ubiquitin–proteasome system, which can be divided in four distinct coupled steps: (1) unfolded protein recognition, (2) translocation across the lipid bilayer, (3) addition and then removal of polyubiquitin, and (4) degradation by the 26S proteasome. The second step takes place through dislocons, aided by adaptors recognizing glycan modifications or misfolded regions. Membrane glycoproteins with lumen-exposed domains are often targeted for ERAD via OS-9/XTP3-B. SEL1L, an ER-resident glycoprotein, bridges substrate recognition factors to the Hrd1 complex [61,62,63,64,65]. Dislocon components like Derlin-1, Derlin-2, AUP1, UBXD8, VIMP, and Herp facilitate this process [66,67,68,69]. SEL1L transfers substrates to Hrd1, an E3 ubiquitin ligase, which targets them for degradation. Dislocon components such as Derlins [70], E3 ligase Hrd1 [62], signal peptide peptidase [71], and Sec61 [72] interact with ERAD substrates for dislocation. Once in the cytoplasm, substrates are ubiquitinated, preventing their return to the ER lumen and increasing their affinity for the p97 complex [73]. Ubiquitin recognition factors and cofactors form complexes with p97, mediating substrate extraction [74,75]. The Hrd1 complex directly recruits p97 to dislocation sites, aiding in substrate capture and translocation to the cytoplasm [62,76]. Finally, gp78 recruits p97 complexes by interacting with p97 [77,78], delocalizing and unfolding the misfolded proteins into the cytoplasm. During the third step, unfolded or misfolded proteins are ubiquitinated in the cytoplasm. Ubiquitin (Ub), a small protein, can form mono- or polyubiquitin chains. This process involves E1, E2, and E3 enzymes, as well as E4 [79,80]. E1 activates Ub in an ATP-dependent manner, transferring it to E2. E2 binds to E3, which promotes ubiquitin transfer to the substrate. E4 further extend the ubiquitin chain. UBA1 is the major E1 enzyme, initiating most ubiquitination reactions [81]. ERAD-related E2 enzymes link ubiquitin to E3 enzymes and participate in the degradation of ERAD components, ensuring quality control. The final step in the ERAD pathway is the degradation of misfolded and ubiquitinated proteins delivered to the 26S proteasome. The 26S proteasome consists of a 20S core particle (CP) and two 19S regulatory particles (RPs). The 19S RP recognizes polyubiquitinated proteins, unfolds them, and controls their entry into the 20S CP(82). The 20S CP hydrolyzes the unfolded protein. After degradation, the proteasome returns to its inactive state [82,83]. The ERAD process is summarized in Figure 2.

When CFTR is fully folded, it is packaged into COPII-coated carriers at ER exit sites (ERESs), which are specialized regions of the ER membrane [84]. These carriers transport the cargo through the ER-to-Golgi intermediate compartment (ERGIC) to the Golgi apparatus, a process orchestrated by COPII coat proteins. The formation of COPII- and COPI-coated vesicles relies on essential components like SAR1 GTPase and the SEC23–SEC24 and SEC13–SEC31 subcomplexes [85]. SAR1 activation enables membrane association [86] and recruitment of Sec16 and Sec23–Sec24 heterodimers [87,88]. Once fully assembled, the COPII coat, comprising SEC13–SEC31 heterodimers polymerized over SAR1–SEC23–SEC24–cargo complexes, induces membrane curvature, leading to vesicle budding [89]. Sec16, an ERES marker, stabilizes SAR1 activation and interacts with Sec12 and COPII components, regulating vesicle formation [90]. The SEC13–SEC31 coat remains on vesicles en route to the Golgi, undergoing phosphorylation cycles that prepare it for vesicle fusion with the Golgi membrane [91]. COPI vesicles manage the retrograde transport of proteins from the Golgi to the ER, which is essential for balancing protein trafficking between these organelles. As CFTR is transported from the early cis-Golgi to the medial cisternae and then to the trans-Golgi, its oligosaccharides linked to Asn894 and Asn900 are modified, causing resistance to endoglycosidase H. These changes increase the apparent molecular weight of CFTR, producing the characteristic mature form (band C), which is never detected in the ER-retained variants p.Phe508del-CFTR [92,93].

At the plasma membrane, CFTR levels are determined by a balance of three processes: anterograde trafficking (where CFTR is delivered from the trans-Golgi network to the PM), endocytosis, and recycling. Both endocytosis and recycling are likely the most important process to regulate the amount of CFTR at the membrane [37].

The most common CF-causing mutation, p.Phe508del, does not induce drastic changes in the molecule but results in a conformational defect [93] that increases its binding to Hsp70, impairs CFTR’s folding, and enhances its degradation [94,95,96]. This mutation disrupts CFTR’s biogenesis at various levels. First, most of the nascent p.Phe508del-CFTR is retained within the ER due to increased interactions with chaperone/co-chaperone complexes such as Hsp70, Hsp90, and co-chaperone Aha1 [12,97,98]. Second, p.Phe508del-CFTR binds to CNX with a higher intensity than normal CFTR, leading to a higher retention of the misfolded p.Phe508del-CFTR in the ER. Third, CFTR folding is linked to its export from the ER to the Golgi, where specific export/retention signals in the protein are recognized. One retention mechanism involves four arginine-framed tripeptides (AFTs) located in the N-terminal tail, NBD1, and RD [99]. The p.Phe508del-CFTR variant fails to exit the ER due to these motifs. However, mutating all four AFTs in p.Phe508del-CFTR permits its escape from ERQC [100]. This rescue does not correct folding but overcomes specific trafficking retention factors. p.Phe508del-CFTR has a reduced stability at the plasma membrane, with a half-life of 1 h compared to wt-CFTR’s, which reaches 3 h in polarized human airway epithelial cells [101]. Co-chaperones and other key factors, such as NHERF-1, Rab11, and the inhibition of Rab-5 endocytosis, play crucial roles in this stability at the plasma membrane [102,103,104].

Thus, p.Phe508del-CFTR presents a conformational defect disrupting the biogenesis of the protein at multiple levels. The mutation increases the binding to chaperones like Hsp70 and Hsp90, enhances the ER retention via interactions with CNX, and prevents proper folding and export due to retention signals. Despite minimal export via COPII-coated vesicles, most of p.Phe508del-CFTR is retained and degraded through ERAD, involving unfolded protein recognition; translocation to the cytoplasm; ubiquitination by E1, E2, and E3 enzymes; and degradation by the 26S proteasome. Even when exported to the Golgi, p.Phe508del-CFTR fails to achieve the glycosylation and structural stability of its wild-type counterpart. At the plasma membrane, its half-life is significantly shorter than that of wild-type CFTR, with stability regulated by co-factors such as NHERF-1, Rab11, and Rab-5. The mutation underscores challenges in CFTR biogenesis, trafficking, and stability, highlighting the importance of targeting molecular mechanisms to improve p.Phe508del-CFTR functionality.

## 3. Unconventional Pathways

Proteins lacking a signal sequence or misfolded proteins can bypass the conventional endoplasmic reticulum (ER)–Golgi-mediated pathway and use the unconventional protein secretion pathway (UPS) [105]. The existence of UPS was suggested by experiments where ER-to-Golgi transport was inhibited by brefeldin A, yet extracellular protein secretion continued, indicating alternative mechanisms [106,107,108].

UPS can be categorized into two types. The first concerns leaderless proteins. This category encompasses pathways like pore-mediated translocation, ABC transporter-based secretion, and autophagy-based secretion. The second concerns proteins with signal peptides or transmembrane domains. In this category, proteins enter the ER but bypass the Golgi apparatus. Type IV UPS falls under this category. While some Type IV UPS proteins may retain ER-acquired high-mannose oligosaccharides, this is not a universal characteristic. These proteins are recognized by specific sorting mechanisms within the ER, which direct them towards UPS.

UPS pathways are stress-induced [13,14], and CFTR (for review, [105]) is one of the increasing number of proteins shown to be able to bypass the Golgi apparatus [15]. Initially, it was observed that CFTR could be transported unconventionally in BHK and CHO cells [109]. Subsequently, it was discovered that ER-to-Golgi blockade or ER stress could induce plasma membrane expression of p.Phe508del-CFTR (band B) via a Golgi bypass trafficking pathway in most cells [10,15]. While the mechanisms are largely unknown, the ER stress-induced pathway is Sar1-independent and transports the band B form of CFTR to the cell surface [15]. Notably, UPS of CFTR is Golgi reassembly stacking protein (GRASP)-dependent [15,16]. UPS is triggered by stress, and both GRASP proteins and HSPs play a role in mediating the stress-induced UPS of CFTR. The transport of p.Phe508del-CFTR to the plasma membrane via UPS likely involves vesicular components associated with autophagy, as GRASP55 is involved in autophagy initiation and autophagosome formation [13,17,110].

The GRASP family, including GRASP65 and GRASP55, plays a crucial role in Golgi organization and function [111,112]. While they are involved in Golgi stacking and membrane tethering, they also participate in UPS, facilitating the secretion of both transmembrane and cytoplasmic proteins under cellular stress [112]. When an ER stress is triggered, the phosphorylation of its serine 441 residue results in its monomerization and re-localization to the ER, where it binds the PDZ domain of specific substrates such as CFTR with its own PDZ domain [15,16,109]. This is likely followed by the encapsulation of the substrate into membrane vesicle carriers that are targeted directly to the plasma membrane in a COPII-independent way using multivesicular bodies (MVBs) [113]. This is supported by the observation that GRASP55 is also involved in autophagosome formation [114]. Therefore, it is plausible that p.Phe508del-CFTR utilizes autophagosome-related vesicles for its unconventional transport to the cell surface.

While autophagy is traditionally viewed as a process for breaking down cellular components, autophagy-related proteins (ATGs) are involved in UPS. Under stress conditions or nutrient deprivation, autophagy functions as a pathway for the transport of specific proteins, particularly membrane proteins [18,105,115,116]. Indeed, a variety of leaderless proteins have been genetically linked to ATG genes for secretion via UPS, including acyl-CoA binding protein, IL-18, HMGB1, and the mutant plasma membrane channel p.Phe508del-CFTR, among others [15,18,117,118]. This unconventional secretory route is referred to as “secretory autophagy” [116]. A selective process known as chaperone-mediated autophagy [119] plays a role here, in which single polypeptides are recognized as unfolded or misfolded when they expose binding sites recognized by the heat shock-cognate protein of 70 kDa (Hsc70) [119,120] or ubiquitins [121]. Autophagy in UPS is characterized by two key features: (1) the involvement of ATG proteins, and (2) its dependence on GRASP55 and GRASP65 in mammalian cells [122]. Mammalian autophagic regulators involved in UPS include ULKs, Beclins, LC3s, and GABARAPs [115].

**Figure 3:**
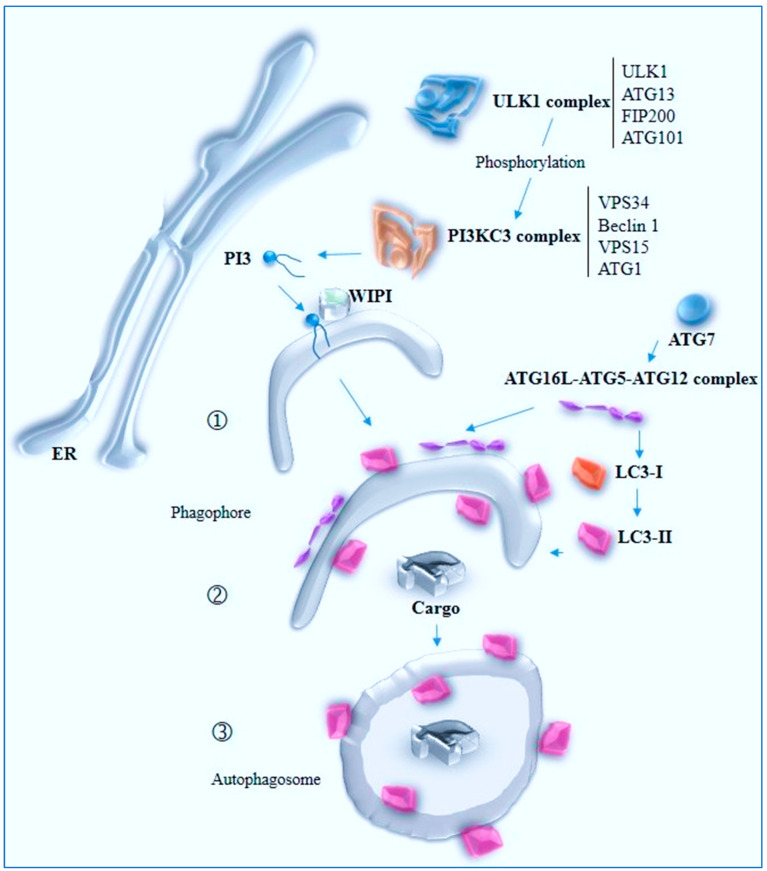
Schematic representation of autophagosome formation The steps are the nucleation of the phagophore (1), its elongation and expansion (2), and its closure (3) to form the double-membrane autophagosome. The ULK complex initiates the process and phosphorylates the PI3KCIII complex, which generates the PI3P lipid-interacting proteins, which recruits proteins such as WIPIs. ATG7 activates ATG12. ATG10 serves as an enzyme transferring ATG12 to ATG5, forming an ATG5–ATG12 conjugate. This conjugate interacts with ATG16L, facilitating its localization to the phagophore. The Atg12–Atg5–Atg16L complex resides on the outer membrane of the phagophore and dissociates from the completed autophagosome. ATG7 also activates LC3, a member of the ATG8 family, which undergoes lipidation, becoming LC3-II, which associates with the phagophore membrane. The LC3-II complex is present on both sides of the phagophore and autophagosome and serves as a marker for autophagosomes and plays a crucial role in cargo recruitment and autophagosome closure. These conjugation systems, thanks to specific enzymes, ensure the proper assembly and expansion of the phagophore.

Autophagosome formation is a multistep process involving various protein complexes [123]. The initial step is nucleation, starting the expansion of the phagophore membrane and leading to autophagosome formation [124]. The ER plays a crucial role in this process through ATG2, a lipid transfer channel [125], and ATG9, a scramblase [126]. ATG9 provides lipid components, while ATG2 bridges the ER and phagophore membranes, transferring newly synthesized lipids. These lipids are rearranged by ATG9 at the phagophore, with the process enabled by the association of ATG2, ATG9, and the WD-repeat domain phosphoinositide-interacting protein (WIPI) [127]. Several key molecular players regulate autophagosome formation. The Unc-51-like kinase (ULK) complex and the class III phosphatidylinositol 3-kinase (PI3K) complex are essential for initiating the process. The ULK complex [128,129], comprising ULK1/2, FIP200, ATG13, and ATG101, is recruited to the autophagosome formation site. The PI3K complex, composed of Vps34, Vps15, Beclin 1, and ATG14, generates phosphatidylinositol 3-phosphate (PI3P), a crucial lipid that acts as an anchor for recruiting additional autophagy proteins such as WIPI1 and the ATG16L1-ATG5-ATG12 complex to the nascent autophagosome [130]. Other important factors include WIPI1, which binds to PI3P and recruits the ATG16L1–ATG5–ATG12 complex. This complex permits the elongation of the phagophore. Meanwhile, LC3, a ubiquitin-like protein, is conjugated to phosphatidylethanolamine (PE) on the autophagosome membrane, forming LC3-II. LC3-II, a key marker for autophagosomes, is essential for their maturation and eventual fusion. Finally, the closure of the phagophore membrane results in the formation of a double-membrane autophagosome (Figure 3).

Following autophagosome formation, the second stage is misfolded protein incorporation. This process selectively targets misfolded or damaged proteins, which are recognized through specific mechanisms involving receptors and adaptors. Indeed, proteins destined for autophagy are often tagged with ubiquitins [121,131] or degenerate KFERQ-like pentapeptide motifs serving as recognition sites for autophagy secretory autophagy receptors (SARs) [131]. SARs such as p62/SQSTM1 and NBR1 simultaneously bind to ubiquitin and autophagy-specific ubiquitin-like modifiers like LC3/GABARAP, which establishes a molecular connection between ubiquitination and autophagy [121,131,132,133]. LIR (LC3-interacting region) motifs in SARs target cargo to the inner surface of the phagophore [120]. p62/SQSTM1, NBR1, and NDP52 possess both a domain that binds to ubiquitin chains and a domain that interacts with the autophagosome membrane through LC3 [132,133]. Autophagosomes are marked by ubiquitin-like ATG8 proteins, which are recruited via covalent lipidation, a process facilitated by the E3-ligase-like ATG16L1 complex. LC3 and its homologs (e.g., GABARAP) are inserted into the autophagosome membrane by conjugation to PE. Autophagy receptors like p62 bind to LC3, tethering target proteins to the membrane of the nascent autophagosome [131,134]. In some cases, target proteins are not directly recognized by autophagy receptors, but adaptors facilitate this recognition by bridging the target protein to autophagy receptors. While these adaptors aid in the selective capture of specific proteins destined for degradation, under certain conditions such as starvation or cellular stress, autophagy can become non-selective, engulfing portions of the cytoplasm and their proteins indiscriminately. In such cases, proteins are not actively targeted or marked but are simply trapped within the forming autophagosome membrane.

The next step is the fusion of the autophagosome with the plasma membrane, which is regulated by SNAREs (v-SNAREs on the autophagosome membrane, t-SNAREs on the plasma membrane) [135]. The fusion forms a SNARE complex, with potential involvement of additional regulatory proteins such as Rab GTPases and tethering factors. After the fusion, the membranes become continuous, allowing membrane proteins to redistribute within the plasma membrane, and the lipid bilayer’s fluidity facilitates the lateral movement of proteins.

Now that we have reviewed the general mechanisms, let us see what happens with our protein of interest, p.Phe508del-CFTR. p.Phe508del-CFTR depends on unconventional protein secretion (UPS) as a rescue mechanism to bypass its inherent folding and trafficking defects. This mutation prevents the protein from folding correctly, leading to its retention in the ER and its subsequent degradation via ERAD. Nevertheless, under conditions of ER stress or ER-to-Golgi blockade, p.Phe508del-CFTR (band B) can bypass the Golgi apparatus and be transported to the plasma membrane through a Sar1-independent pathway. GRASP55 is a critical mediator of this UPS pathway, facilitating the transport of p.Phe508del-CFTR to the plasma membrane. Under stress conditions, GRASP55 is phosphorylated at serine 441, causing it to monomerize and relocate to the ER. There, it interacts directly with p.Phe508del-CFTR through its PDZ domain, contributing to the protein’s unconventional trafficking. This bypass route allows p.Phe508del-CFTR to reach the cell surface without passing through the Golgi apparatus, distinguishing it from the conventional CFTR trafficking pathway. Additionally, autophagy-related mechanisms contribute to the unconventional secretion of p.Phe508del-CFTR, as stress-induced autophagy pathways can play a supportive role in its trafficking. Proteins such as ATG2, ATG9, LC3, and GABARAPs regulate autophagic processes and are crucial for maintaining cellular homeostasis during protein transport. Autophagy receptors like p62/SQSTM1 and NBR1 recognize ubiquitinated p.Phe508del-CFTR, further aiding in the regulation and trafficking of the mutant protein [132,133,134]. These mechanisms ensure selective and efficient handling of p.Phe508del-CFTR, facilitating its delivery to the plasma membrane under specific conditions.

The ESCRT machinery also plays an important role in the unconventional trafficking of p.Phe508del-CFTR. The ESCRT-I complex protein MVB12B has been shown to be critical for the process, as its depletion disrupts the mutant protein’s ability to reach the plasma membrane. Conversely, overexpression of MVB12B restores partial plasma membrane expression and ion channel function of p.Phe508del-CFTR, highlighting its significance in the UPS pathway [18]. In the final stages of UPS, p.Phe508del-CFTR utilizes cellular machinery to reach and integrate into the plasma membrane. Sec16A (SEC16 homolog A, endoplasmic reticulum export factor), a key protein in UPS, relocates to the cell periphery during ER stress and interacts with GRASP55 to enhance the trafficking of p.Phe508del-CFTR. Indeed, in addition to GRASP55, the UPS of pPhe508del-CFTR also involves Sec16A [15,39]. During ER stress, Sec16A moves to the cell periphery and binds GRASP55. In contrast, Sec31, a key COPII component, stays near the microtubules. This indicates that Sec16A operates independently of the core COPII components. Sec16A’s peripheral localization could enhance UPS by reducing the distance between the ER exit site and the plasma membrane [39]. The levels of Sec16A and the UPS of p.Phe508del-CFTR are regulated by IRE1, a transducer of the UPR [15]. Therefore, UPS, the UPR, and autophagosome formation are involved in the membrane transport of p.Phe508del-CFTR to the membrane. The implication of autophagosomes is reinforced by the involvement of the endosomal sorting complex required for transport (ESCRT) in the unconventional trafficking of core-glycosylated CFTR [17]. This process operates independently of conventional COPII components and is regulated by the unfolded protein response (UPR) via IRE1 [16,136]. Together, GRASP55, autophagy-related pathways, and UPR signaling ensure that p.Phe508del-CFTR bypasses conventional trafficking barriers and reaches the cell surface.

These pathways collectively highlight the reliance of p.Phe508del-CFTR on UPS and autophagy to overcome its folding and trafficking defects. By exploiting these mechanisms, the mutant protein can bypass conventional routes, partially restoring its surface expression and function, thus offering potential therapeutic insights for cystic fibrosis treatment.

## 4. UPR Regulates the Autophagy-Mediated UPS of p.Phe508del-CFTR

The unfolded protein response (UPR) not only triggers UPS but also induces autophagy through its effectors, IRE1, PERK, and ATF6. Each of these UPR pathways contributes to autophagy-mediated mechanisms, which are crucial for the UPS of p.Phe508del-CFTR.

During the UPR, the IRE1 sensor dimerizes and undergoes autophosphorylation, activating its RNase activity [136]. This activation results in the splicing of the mRNA of the transcription factor X-box-binding protein 1 (XBP-1), where a 26-nucleotide intron is removed. This splicing introduces a frameshift, producing the spliced XBP-1 (XBP-1s) transcript. XBP-1s is translated into a transcription factor that upregulates genes involved in protein folding, quality control, ER-associated degradation (ERAD), lipid metabolism, and pro-inflammatory responses [137,138,139,140]. IRE1–XBP1s signaling also induces autophagy through several mechanisms. XBP-1s regulates Bcl-2 expression, promoting autophagy [141,142]. Additionally, XBP-1s enhances autophagy by increasing LC3-I-to-LC3-II conversion and beclin-1 expression. XBP-1s directly binds to the BECN1 promoter as a homodimer, further enhancing beclin-1 transcription. Another pathway involves IRE1 phosphorylation of JNK, a stress-related protein kinase of the MAPK family [143,144]. The IRE1/TRAF2/JNK1 axis activates autophagy by promoting the formation of autophagic vacuoles and LC3-positive vesicles. JNK1 directly regulates beclin-1 expression and phosphorylates ER-localized Bcl-2, causing the release of beclin-1 from its inhibitory interaction with Bcl-2. Both the IRE1/XBP1s and IRE1/JNK1 pathways converge on beclin-1, an essential ATG protein involved in vesicle nucleation.

The activation of the serine/threonine kinase PERK involves its autophosphorylation and homodimerization. PERK then phosphorylates eukaryotic translation initiation factor 2 alpha (eIF2α), which promotes the translation of activating transcription factor 4 (ATF4), a critical UPR mediator of autophagy [145]. PERK/eIF2α activation promotes vesicle formation and LC3-I-to-LC3-II conversion, key steps in autophagy induction [145,146]. ATF4 also enhances the expression of ATG12, an essential protein for autophagosome elongation [147]. Moreover, ATF4 binds to the cAMP response element in the LC3β promoter, increasing its transcription and further promoting autophagy [148]. ATF4 activation leads to the expression of CHOP, which regulates autophagy by increasing ATG5- and BH3-only protein expression while reducing Bcl-2 levels. This action further releases beclin-1, facilitating autophagy. CHOP also induces the expression of pro-apoptotic BH3-only proteins, such as Bim and Puma, which enhance beclin-1 activity. Furthermore, the eIF2α/ATF4/CHOP axis increases p62 expression, a key regulator of autophagy [149].

ATF6 functions as an ER membrane sensor with its C-terminal domain in the ER lumen and its N-terminal transcription factor domain in the cytosol [29]. Under conditions of ER stress, ATF6 dissociates from GRP78, exposing its Golgi localization signals, which facilitate its transport to the Golgi apparatus. In the Golgi, ATF6 undergoes cleavage by site-1 and site-2 proteases [150]. The cleaved, active ATF6 is then translocated to the nucleus, where it binds ER stress-associated elements and induces the expression of GRP78, GRP94, XBP1, CHOP, and protein disulfide isomerase (PDI), which are essential for proper protein folding and secretion [137,151,152]. ATF6 is also crucial for autophagy induction. It upregulates death-associated kinase 1 (DAPK1), which is involved in autophagosome formation via beclin-1 phosphorylation. Additionally, ATF6-mediated upregulation of CHOP, XBP1, and GRP78 contributes to autophagy induction.

Whereas the activation of UPR due to the retention of p.Phe508del-CFTR in the ER is still under debate, it has been suggested that recombinant p.Phe508del-CFTR activates the UPR, leading to the inhibition of endogenous CFTR expression (32,155–157). It is suggested that high levels of p.Phe508del-CFTR retention are necessary to trigger this pathway [32,153,154,155]. The upregulation of IRE1α mRNA levels in freshly isolated CF human bronchial epithelial cells compared to normal epithelial cells is another indication of UPR activity in CF cells [156]. While the IRE1α/XBP-1 branch of the UPR is proposed to be triggered by inflammation in CF airway epithelia [139], IRE1α mRNA levels appear elevated in CF cells before the onset of inflammation [157], suggesting that the UPR may occur prior to inflammatory responses. Finally, it is hypothesized that the UPR is triggered when p.Phe508del-CFTR retention in the ER exceeds a threshold in link with ERAD capacity. Above a certain limit, the UPR activates transcription of chaperones and ERAD proteins. This threshold may be influenced by factors such as ER overload by other proteins, including inflammatory proteins, or impaired ERAD function. This idea is supported by ratiometric sensing of BiP-client versus BiP levels [158,159]. Regardless of the exact triggering, the UPR is observed in CF cells and plays a crucial role in the disease’s pathophysiology. Therefore, the involvement of the UPR when p.Phe508del-CFTR is expressed regulates the autophagy-mediated UPS of the protein.

## 5. Evidence Supporting the Autophagosome-Mediated UPS of p.Phe508del-CFTR

Several pieces of evidence suggest that the UPS of p.Phe508del-CFTR relies on autophagosome-mediated mechanisms. CFTR utilizes UPS through GRASP55, a protein known to play a role in autophagy-dependent mechanisms [160,161]. Furthermore, components involved in autophagosome formation and the ESCRT machinery are essential for the UPS of core-glycosylated CFTR [17]. The ESCRT machinery also cooperates with SNARE proteins during autophagosome maturation [162] and is critical for the formation of multivesicular bodies (MVBs), which encapsulate ubiquitin-tagged proteins. Additionally, the ESCRT-I complex protein MVB12B is vital for the UPS of p.Phe508del-CFTR. Depletion of MVB12B disrupts the trafficking of the mutant protein to the cell surface, whereas its overexpression partially restores surface expression and ion channel function [17]. Furthermore, a reduction in autophagy-related proteins such as ATG5 and ATG7 inhibits the secretion of core-glycosylated CFTR, further supporting the involvement of autophagosomes in this process [17].

## 6. Discussion and Conclusions

**Figure 4:**
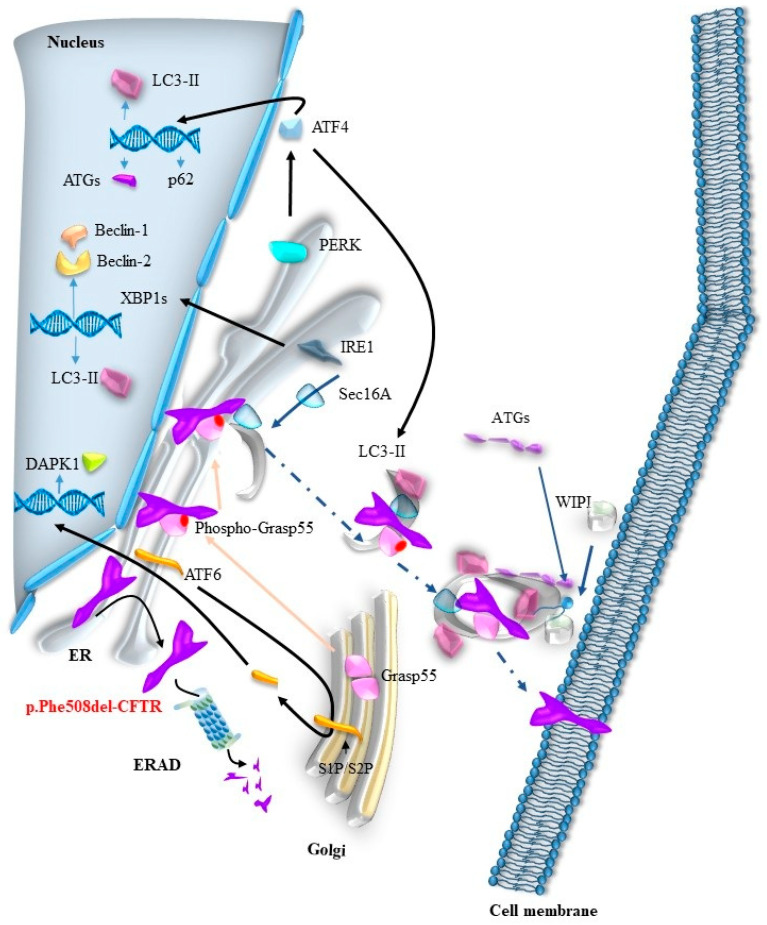
The route of p.Phe508del-CFTR from the ER to the membrane in stressed cells. A stress such as the UPR activates the three effectors IRE1, PERK, and ATF6 (black arrows), which are involved in the formation of autophagosomes through the overexpression of LC3-I and LC3-II, p62, ATG2, DAPK1, and Beclin-1 and -2. The newly synthesized proteins then take part in the formation of phagosomes (blue arrows). Concurrently, GRASP55, monomerized by phosphorylation, migrates to the ER, where it binds to p.Phe508del-CFTR via the PDZ domains (yellow arrow). Sec16a, induced by IRE1, links GRASP55 to the ER membrane by initiating the formation of a membrane vesicle (independent of COPII in the case of CFTR). All the necessary elements for the formation of an autophagosome are then recruited, as described earlier in the text. Finally, the autophagosome containing p.Phe508del-CFTR fuses with the plasma membrane, releasing the channel into the lipid bilayer of the membrane.

The p.Phe508del-CFTR protein can be transported to the cell membrane through the unconventional protein secretion (UPS) pathway, using GRASP55 as a carrier. This process is particularly relevant under cellular stress conditions, such as the unfolded protein response (UPR). However, the specific mechanisms underlying the transport of the complex formed by p.Phe508del-CFTR and phosphorylated GRASP55 from the endoplasmic reticulum (ER) to the plasma membrane remain insufficiently described. Given the established connections between UPS and autophagy, and the links between the UPR and autophagosome formation, we propose that the p.Phe508del-CFTR-GRASP55 complex may utilize autophagosomes for its transport to the membrane, as depicted in Figure 4.

The transport of membrane proteins involves a highly intricate network of pathways, including conventional protein secretion (CPS), UPS, and autophagy systems. These pathways collectively contribute to maintaining cellular homeostasis. This review emphasizes the interconnected roles of UPS and autophagy in ensuring the proper transport of membrane proteins, such as p.Phe508del-CFTR, particularly under stress conditions. Understanding these mechanisms is crucial not only for advancing basic cellular biology but also for informing the development of targeted therapeutic strategies. The decision-making process determining whether a protein follows CPS or UPS pathways depends on a combination of factors, including the presence of specific signal sequences, the intrinsic properties of the protein, and the cellular environment, such as stress conditions. Proteins that lack a signal peptide often utilize UPS pathways, which may involve autophagosomes for transport. Transmembrane proteins can also follow UPS depending on the physiological and cellular context. This choice is influenced by the cell type, its functional needs, and its capacity for CPS or UPS. The UPR plays a pivotal role in these decisions. It enhances the ER’s capacity to manage misfolded proteins by upregulating chaperones and proteins involved in folding. This allows proteins destined for CPS to fold properly and proceed through the ER–Golgi pathway. If the UPR cannot resolve the folding imbalance, it initiates alternative pathways, including UPS and autophagy, to alleviate cellular stress. Notably, the UPR can independently activate autophagy, promoting protein insertion into membranes.

Disruptions in transport pathways, particularly under conditions of ER stress, are associated with various diseases. These diseases can be grouped into two distinct categories:

The first category includes diseases linked to protein alterations, where the primary issue lies with the proteins themselves, such as misfolding or altered glycosylation, rather than defects in the secretory machinery itself. In the case of CF, mutations in the *CFTR* gene, such as p.Phe508del, result in misfolded proteins that fail to efficiently reach the plasma membrane. Neurodegenerative diseases, such as Alzheimer’s and Parkinson’s, are characterized by the accumulation of misfolded proteins, including amyloid-β and α-synuclein [163,164]. In Alzheimer’s disease, autophagy can help clear protein aggregates, such as amyloid-β and tau, but excessive autophagy can also contribute to neuronal damage [164,165]. Dysregulation of autophagy and ER stress can contribute to neuronal degeneration in amyotrophic lateral sclerosis [164]. Other proteins, such as polycystin-2 and mutant Mpl, also rely on UPS for trafficking to the cell surface [166,167]. Modulating these pathways could enhance the surface expression of mutant proteins, offering potential therapeutic benefits.

The second category includes diseases arising from defects in UPS or autophagy components, where the dysfunction lies within the pathways themselves. UPS and autophagy are crucial for cellular stress responses, including ER stress, and their dysregulation can contribute to various pathologies. Proteins such as beclin-1 and ATGs play pivotal roles in these pathways. Beclin-1, for example, regulates both autophagy and apoptosis, and its modulation significantly influences cellular survival and death. This is particularly important in diseases like cancer and cardiac dysfunction [168]. Mono-allelic deletions of the BECN1 gene are found in up to 50% of breast cancers, 75% of ovarian cancers, and 40% of prostate cancers [141,169,170]. Mutations in the ATG genes are also responsible for various pathologies. Mutations in the ATG9A gene disrupt the autophagy process, thereby promoting the development of cancers. In the case of breast cancer, particularly in triple-negative patients (cancers that do not present estrogen, progesterone, or HER2 receptors), these mutations worsen disease progression [171]. Mutations in the ATG5 gene have been linked to neurodegenerative diseases [172], and mutations in the ATG7 gene have been associated with lipid metabolism disorders [173].

Targeting UPS and autophagy presents promising therapeutic avenues. By modulating these pathways, it may be possible to enhance the secretion of therapeutic proteins; suppress the secretion of harmful proteins such as inflammatory cytokines, which could benefit neurodegenerative and inflammatory disorders; and exploit UPS to deliver therapeutic agents directly to target cells. However, several critical questions remain unanswered, which require further investigation. One such question is the identification of the specific carriers involved in non-vesicular UPS. Additionally, the types of vesicles that participate in vesicular UPS and the roles they play in cargo transport need to be better understood. Finally, it remains unclear what distinguishes transmembrane proteins that follow UPS from those that utilize the conventional secretory pathway. Addressing these questions could provide valuable insights for the development of targeted therapies in the future.

This review presents an overview of the key mechanisms of protein quality control, highlighting their importance in the management of misfolded proteins. Although mutated proteins are often studied in isolation, we find it crucial to place p.Phe508del-CFTR within the broader context of cellular stress responses. Understanding its regulation by GRASP55, the switch between degradative and secretory autophagy, and the role of membrane particles in UPS could open new therapeutic opportunities. Additionally, elucidating the molecular distinctions between CPS and UPS pathways will be essential for developing targeted treatments for diseases involving defective protein transport.

In summary, we described how p.Phe508del-CFTR is rescued through the UPS pathway, occurring under stress conditions such as ER stress, which activate alternative transport mechanisms. UPS employs key proteins, including GRASP55, which interacts directly with p.Phe508del-CFTR, facilitating the mutant protein’s transport to the plasma membrane through a Sar1-independent mechanism. Additionally, autophagy-related pathways support this process. Proteins like ATGs, LC3, and GABARAP regulate autophagosome formation, ensuring cellular homeostasis during protein trafficking. Autophagy receptors (p62/SQSTM1 and NBR1) recognize ubiquitinated p.Phe508del-CFTR and tether it to autophagy-specific proteins for unconventional transport. The ESCRT machinery facilitates autophagosome maturation, enabling the mutant protein to bypass the Golgi apparatus and reach the plasma membrane. Finally, Sec16, regulated by the UPR via IRE1, enhances UPS by relocating to the cell periphery, bridging ER exit sites with plasma membrane transport.

Why is it essential for p.Phe508del-CFTR to be rescued through UPS? The rescue of p.Phe508del-CFTR by UPS is necessary because the mutation prevents proper folding, causing the protein to be retained in the ER and targeted to ERAD. Conventional ER–Golgi transport pathways cannot process p.Phe508del-CFTR due to its structural instability and retention signals. UPS provides an alternative route that bypasses these defects, allowing the mutant protein to avoid ERAD and reach the plasma membrane, even in its immature, core-glycosylated band B form. UPS is triggered because conventional mechanisms fail, and cellular stress responses, such as the UPR, prioritize alternative pathways to rescue dysfunctional proteins like p.Phe508del-CFTR. This process is an alternative to a process that cannot proceed, offering a second chance for p.Phe508del-CFTR to reach the plasma membrane. It can be likened to a lifeline, enabling the mutant protein to achieve its functional destination despite its structural defects.

While UPS, the UPR, and autophagy pathways are highly complex and interconnected, their modulation represents a promising strategy for addressing a wide range of diseases. Further research into the molecular details of these pathways will be critical for unlocking their therapeutic potential. Triggering UPS and autophagy to improve the trafficking and rescue of p.Phe508del-CFTR represents a promising approach to finding new therapeutic strategies for CF. These pathways can help bypass the conventional ER–Golgi route, addressing the inherent folding and trafficking defects of the mutant protein. However, modulating these quality control systems presents numerous challenges that must be carefully considered to balance therapeutic potential with safety and specificity. One major challenge lies in the complexity and central roles of the mechanisms involved. Both UPS and autophagy are integral to cellular homeostasis, managing protein folding, degradation, and trafficking. Modulating these pathways for p.Phe508del-CFTR rescue could inadvertently disrupt their normal functions, leading to unintended effects. For example, autophagy, while beneficial for unconventional CFTR trafficking, also plays a role in the degradation of cellular components and damaged organelles. Overactivation may lead to excessive degradation, compromising cell viability, while underactivation might hinder the clearance of toxic protein aggregates or dysfunctional organelles. Another concern is the specificity of targeting these pathways. Proteins like GRASP55, which play a key role in the UPS of p.Phe508del-CFTR, also contribute to essential processes such as Golgi stacking and membrane tethering. Modulating GRASP55 or related autophagy regulators could interfere with these critical cellular functions, potentially causing broader disruptions. Similarly, the ESCRT machinery, crucial for autophagosome maturation, is also involved in numerous cellular processes, from endocytosis to membrane repair. Nonspecific effects on these systems could exacerbate rather than alleviate disease symptoms. Furthermore, the therapeutic safety of altering stress response pathways such as the UPR must also be considered. Its activation, which regulates key players like Sec16A in the UPS of p.Phe508del-CFTR, is part of the broader ER stress response. Excessive modulation could result in prolonged ER stress, potentially triggering apoptosis and worsening cellular dysfunction. Similarly, reducing the activity of retention signals or ERAD could lead to the escape of not just p.Phe508del-CFTR but also other misfolded or non-functional proteins, increasing the risk of proteotoxic stress. The functional rescue of p.Phe508del-CFTR itself poses further challenges. Even when delivered to the plasma membrane via UPS, the mutant protein may remain less stable and have a shorter half-life compared to wild-type CFTR. Post-trafficking mechanisms, such as endocytosis and recycling, could also be modulated to ensure sustained surface expression and functionality of the rescued protein. This highlights the need for a multifaceted therapeutic approach that not only enhances delivery but also addresses long-term stability and activity. From a translational perspective, the development of small-molecule modulators for these pathways is promising but must be approached with caution. Achieving selective modulation of UPS and autophagy for p.Phe508del-CFTR without off-target effects remains a significant hurdle. Broad-spectrum modulators risk disrupting other proteins or pathways, while overly specific agents may require precise patient stratification, complicating their clinical application.

Despite these challenges, the therapeutic potential of triggering UPS and autophagy remains compelling. Leveraging these pathways could provide alternative solutions for patients with limited responses to current therapies like CFTR modulators. To mitigate risks, a combination therapy approach might be effective, pairing modulators that enhance p.Phe508del-CFTR trafficking with stabilizers that prolong its functional presence at the membrane. Additionally, further research into the molecular mechanisms of UPS and autophagy will be critical for identifying novel targets and refining therapeutic strategies.

In conclusion, while the modulation of UPS and autophagy for p.Phe508del-CFTR rescue presents significant challenges, it also offers exciting opportunities to advance CF treatment. A careful approach is essential to harnessing these mechanisms safely and effectively, ensuring that the benefits outweigh the risks for patients.

## Data Availability

No new data were created or analyzed in this study.

## Abbreviations

ATF6	Activating transcription factor 6
ATGs	Autophagy-related proteins
CNX	Calnexin
CRT	Calreticulin
CFTR	Cystic fibrosis transmembrane conductance regulator
CPS	Conventional protein secretion
CF	Cystic fibrosis
ER	Endoplasmic reticulum
ESCRT	Endosomal sorting complex required for transport
ERAD	ER-associated degradation
EDEM	ER degradation-enhanced mannosidase
ERES	ER exit sites
ERQC	ER quality control
GA	Golgi apparatus
GRASP	Golgi reassembly stacking protein
IRE1	Inositol-requiring enzyme 1
LC3	Light chain 3
LIR	LC3-interacting region
NEFs	Nucleotide-exchange factors
OST	Oligosaccharyltransferase
PI3P	Phosphatidylinositol 3-phosphate
PDI	Protein disulfide isomerase
PERK	Protein kinase R (PKR)-like endoplasmic reticulum kinase
SARs	Secretory autophagy receptors
SP	Signal peptide
SRP	Signal recognition particle
SNAREs	Soluble N-ethylmaleimide-sensitive-factor Attachment protein Receptors
TRAP	Translocon-associated protein
UPS	Unconventional protein secretion
UPR	Unfolded protein response
UDP	Uridine diphosphate
WIPI	WD-repeat domain phosphoinositide-interacting protein
